# The development and clinical application of a novel schizophrenia screening system using yoga-induced autonomic nervous system responses

**DOI:** 10.3389/fphys.2022.902979

**Published:** 2022-10-05

**Authors:** Tomoko Inoue, Toshikazu Shinba, Masanari Itokawa, Guanghao Sun, Maho Nishikawa, Mitsuhiro Miyashita, Kazuhiro Suzuki, Nobutoshi Kariya, Makoto Arai, Takemi Matsui

**Affiliations:** ^1^ Graduate School of Systems Design, Tokyo Metropolitan University, Hachioji, Japan; ^2^ Schizophrenia Research Project, Department of Psychiatry and Behavioral Sciences, Tokyo Metropolitan Institute of Medical Science, Tokyo, Japan; ^3^ Department of Psychiatry, Tokyo Metropolitan Matsuzawa Hospital, Tokyo, Japan; ^4^ Department of Psychiatry, Shizuoka Saiseikai General Hospital, Shizuoka, Japan; ^5^ Graduate School of Informatic and Engineering, The University of Electro-Communications, Chofu, Japan; ^6^ Department of Psychiatry, Takatsuki Clinic, Akishima, Japan; ^7^ Kokoro-no Home Clinic, Tokyo, Japan; ^8^ Maynds Tower Mental Clinic, Tokyo, Japan

**Keywords:** schizophrenia, autonomic nervous, heart rate variability, yoga, screening, sensitivity, mindfulness, wearable device

## Abstract

**Background:** In severe cases, schizophrenia can result in suicide and social isolation. Diagnosis delay can lead to worsening symptoms, and often results in prolonged therapy. An estimated 50%–80% of patients with schizophrenia are unaware of their condition. Biomarkers for schizophrenia are important for receiving a diagnosis from a psychiatrist at an early stage. Although previous studies have investigated near-infrared spectroscopy as a biomarker for schizophrenia, the required equipment is expensive and not designed for home use. Hence, we developed a novel home-use schizophrenia screening system that uses a wearable device to measure autonomic nervous system responses induced by yoga, which is frequently adopted in rehabilitation for schizophrenia.

**Materials and methods:** The schizophrenia screening system automatically distinguishes patients with schizophrenia from healthy subjects *via* yoga-induced transient autonomic responses measured with a wearable wireless electrocardiograph (ECG) using linear discriminant analysis (LDA; Z score ≥ 0 → suspected schizophrenia, Z-score < 0 → healthy). The explanatory variables of LDA are averages of four indicators: components of heart rate variability (HRV): the very low-frequency (VLF), the low-frequency (LF), HR, and standard deviation of the NN intervals (SDNN). In the current study, HRV is defined as frequency domain HRV, which is determined by integrating RRI power spectrum densities from 0.0033 to 0.04 Hz (VLF) and 0.04–0.15 Hz (LF), and as time domain HRV, SDNN of which is calculated as the mean of the standard deviations of the RR intervals. These variables were measured before (5 min), during (15 min), and after (5 min) yoga in a 15-min mindfulness-based yoga program for schizophrenia (MYS). The General Health Questionnaire-28 (GHQ28) score was used to assess the severity of mental disorders for patients with schizophrenia and healthy volunteers. Twelve patients with schizophrenia (eight female and four male, 23–60 years old) and 16 healthy volunteers (seven female and nine male, 22–54 years old) were recruited.

**Results:** The schizophrenia screening system achieved sensitivity of 91% and specificity of 81%. Z-scores of LDA were significantly correlated with GHQ28 scores (*r* = 0.45, *p* = 0.01).

**Conclusion:** Our proposed system appears to be promising for future automated preliminary schizophrenia screening at home.

## Introduction

Schizophrenia is an intractable mental illness that most frequently develops during adolescence, and has a prevalence rate of approximately 1%. In its early stages, schizophrenia can be difficult to diagnose without patients sharing their subjective symptoms, such as hallucinations, which frequently go undetected by both patients and psychiatrists. Previous studies have reported that there is no recognition of symptoms in 50%–80% of patients in the early stage of schizophrenia (Gharabawi et al., 2006; Raffard et al., 2008), which can result in a long duration of untreated psychosis (DUP) and nonadherence (Dassa et al., 2010; Kaminga et al., 2019). Early detection of schizophrenia is essential for improving outcomes, such as shorter recovery, maintenance of social functioning, and less hospital stays. On the contrary, prolonged DUP can lead to more severe symptoms (Perkins et al., 2005), poor recovery, social dysfunction (Marshall et al., 2005), and increased suicidality rate (Barrett et al., 2015). It is therefore important to develop screening methods for early detection of schizophrenia.

Biomarkers for schizophrenia are important for receiving a diagnosis from a psychiatrist at an early stage. Previous studies have investigated methods for detecting various potential biomarkers for schizophrenia, such as near-infrared spectroscopy (NIRS) (Koike et al., 2014) and exploratory eye movement (EEM) testing (Suzuki et al., 2009). However, these schizophrenia biomarker systems are expensive, and are not designed for home use. Using a wearable device involving a smartwatch, we developed a novel home-use schizophrenia screening system by measuring yoga-induced autonomic transient responses while patients watched a yoga instruction video (https://youtu.be/c4xzmA22h84). We adopted a yoga task because some schizophrenia patients have difficulty performing conventional mental tasks, such as random number generation. Promising effects of yoga on schizophrenia patients have been reported (Sabe et al., 2019; Schulze et al., 2021).

In our previous work, we developed a major depressive disorder (MDD) screening system that uses transient autonomic responses induced by a random number generation task to screen for MDD (Sun et al., 2016; Dagdanpurev et al., 2018). However, schizophrenia patients who experience stress vulnerability cannot adequately conduct the random number generation task in a time-pressured situation.

In addition, previous studies have revealed that patients with schizophrenia exhibit vagal tone reduction in the resting state, which is correlated with schizophrenia severity (Bär et al., 2005; Montaquila et al., 2015). However, during rest, parasympathetic activity reductions vary greatly between individuals with schizophrenia, which makes it difficult to use resting-state parasympathetic activity as a diagnostic biomarker. Instead, we adopted yoga-induced autonomic responses as a screening means. Most patients were able to practice yoga in this study protocol because it only required them to make simple movements while watching a yoga instruction video. For patients with schizophrenia, yoga appears to be favorably accepted by patients compared with numerical tasks such as a random number generation task.

Autonomic activity data were acquired using a wearable device before, during, and after yoga sessions. We performed linear discriminant analysis (LDA) of the autonomic function-related explanatory variables of very low-frequency (VLF), low-frequency (LF) for frequency-domain parameters, standard deviation of the NN intervals (SDNN) for time domain parameters and HR to distinguish patients with schizophrenia from healthy volunteers. Here, we report a clinical test of the proposed screening system, which included 12 patients with schizophrenia and 16 healthy control participants. The proposed schizophrenia screening system does not require psychiatrist-recorded patient medical history, and could hold promise for the development of home-based schizophrenia screening methods.

## Materials and Methods

### Participants

From 29th September 2017 to 5th June 2021, 12 schizophrenia outpatients (eight female, four male, 23–60 years old) who were diagnosed according to the schizophrenia criteria of the Diagnostic and Statistical Manual of Mental Disorders (DSM-5) were recruited from Tokyo Metropolitan Matsuzawa Hospital, Takatsuki Clinic, Kokoro-no Home Clinic, and Maynds Tower Mental Clinic. Patients with schizophrenia were recruited by psychiatrists, with exception of ones with comorbid psychiatric disorders such as developmental disorders, mood disorders, and ones with previous or ongoing treatment for cardiac diseases such as angina pectoris and myocardial infarction, and arrhythmias. Healthcare professionals including certificated yoga therapist verified that all the procedures were conducted properly. In addition, from 10th October 2018 and 21st April 2021, 16 healthy volunteers (seven female, nine male, 22–54 years old) were recruited at the Tokyo Metropolitan Institute of Medical Science, Tokyo Metropolitan University.

General Health Questionnaire-28 (GHQ28) scores were used to assess the severity of mental disorder for schizophrenia patients and healthy volunteers. Participants were asked not to consume alcohol or coffee for 24 h prior to the study. We obtained written informed consent from all participants. This study was conducted in accordance with the International Conference on Harmonisation Regulations and was approved by the Ethics Committees of the Tokyo Metropolitan Institute of Medical Science (approval number 19-21), Tokyo Metropolitan University (approval number H21-014) and Tokyo Metropolitan Matsuzawa Hospital (approval number 2019-4). All research organizations were in Tokyo, Japan.

### Measurement protocol

**FIGURE 1:**
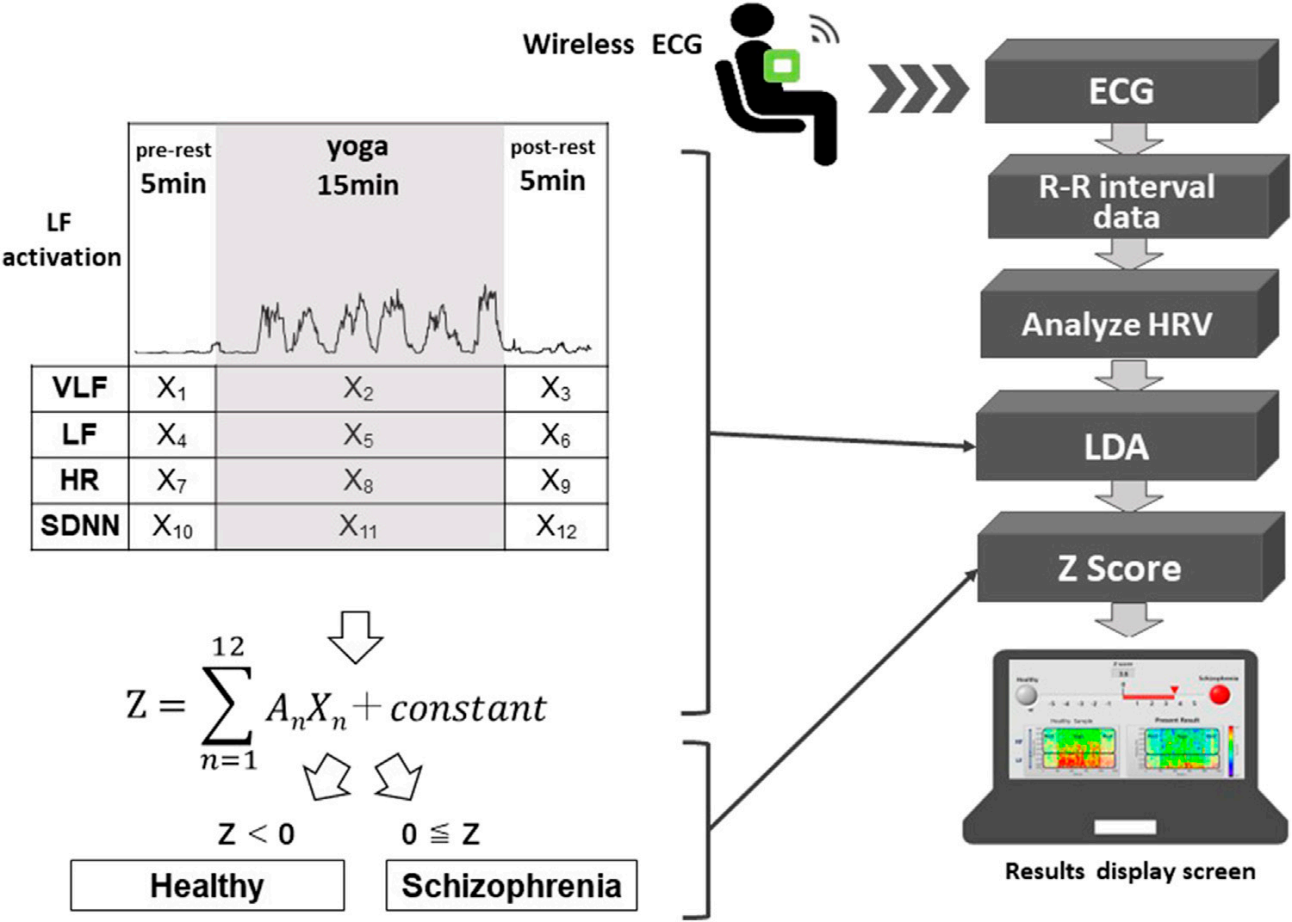
Schematic diagram of the proposed system. The system automatically distinguishes possible schizophrenia cases from healthy people using autonomic nervous system activity-related explanatory variables (X_1_ to X_12_) and regression coefficients (A_1_ to A_12_) of an LDA corresponding to the pre-rest, during yoga, and post-rest periods. X_2_, X_5,_ X_8_, and X_11_ are explanatory variables during yoga, which correspond to VLF, LF, HR and SDNN, respectively. The system classifies a user as having suspected schizophrenia when the Z-score of the LDA is greater than or equal to zero.

A wearable wireless ECG was used to measure autonomic nervous system activity in schizophrenia and healthy participants while sitting in a chair before (resting state for 5 min), during (yoga practice for 15 min), and after practicing yoga (resting state for 5 min), as shown in Figure 1. GHQ28 scores were measured prior to autonomic nervous system activity measurements.

### System development

A schematic diagram of the system is shown in Figure 1. Using a wearable wireless ECG monitor (GM3, RF-ECG2), unipolar precordial lead ECG (sampling rate = 256 Hz) was monitored before, during, and after yoga practice. The unipolar precordial lead ECG data were transferred to a personal computer through a wireless ECG receiver with a USB port. The data processing program was written using LabVIEW graphical block diagram programming language (National Instruments, Texas, United States). ECG R-wave peak detection was conducted to determine time series of RR intervals (RRIs). To evaluate autonomic nervous activation induced by yoga, HRV was calculated using fast Fourier transform (FFT; LF and HF) and maximum entropy method (MEM; VLF) from the RRI time series. SDNN and root mean square of successive differences (RMSSD) were used as time domain analyses to evaluate changes in RR intervals (Malik et al., 1996). LDA was conducted to evaluate the Z-scores using VLF, LF, HR, and SDNN components of HRV, before, during, and after yoga practice. The proposed system classifies an examinee as having suspected schizophrenia when the Z-score is greater than or equal to zero.

**FIGURE 2:**
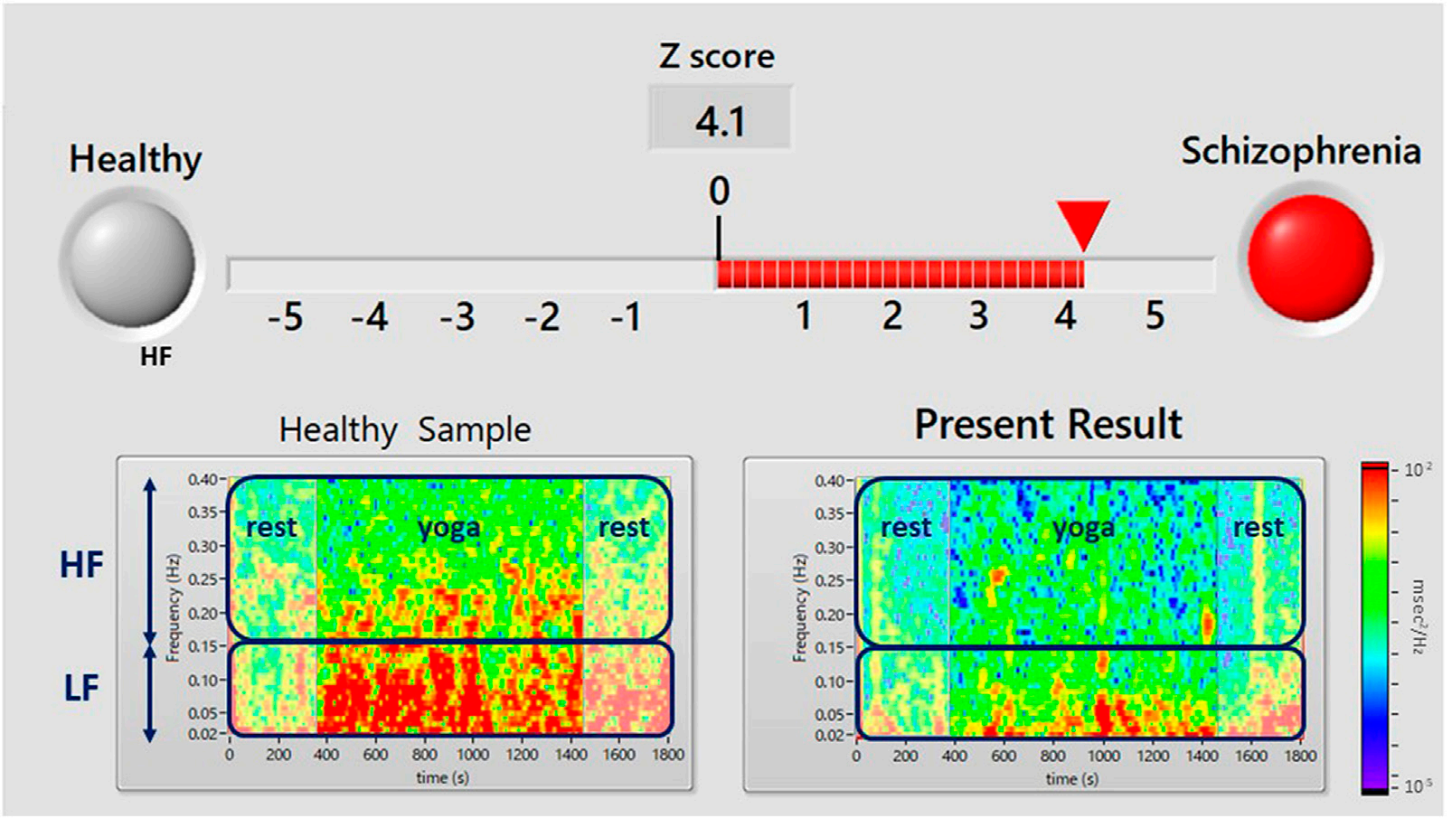
Results display screen of the proposed system. A flashing red light (upper right) indicates that the user has suspected schizophrenia (i.e., a Z score ≥ 0). The Z-score is calculated from yoga-induced autonomic responses using LDA. The color map in the lower panel shows the power spectrum density of HRV during the pre-rest, during yoga, and post-rest periods. The upper and lower squares show the power spectrum densities corresponding to the LF (0.04–0.15 Hz) component of HRV, which is associated with sympathetic and parasympathetic nervous activity, and the HF (0.15–0.4 Hz) component of HRV, which is associated with parasympathetic nervous activity. The lower left panel shows an example of a healthy person’s color map and the right panel shows a color map of the present examinee.

An example of the display screen of the proposed system is shown in Figure 2. A red signal (upper right, representing an examinee that is suspected to have schizophrenia) appears when the Z-score is greater than or equal to zero, and a red bar is an indicator of the Z-score. A green signal (upper left, showing an examinee who appears to be mentally healthy) appears when the Z-score is less than zero. A color map (lower right) shows the yoga-induced autonomic nervous responses expressed in power spectrum densities corresponding to the LF (0.04–0.15 Hz) and HF (0.15–0.4 Hz) domains. For comparison, a typical color map of a healthy volunteer is also shown in the lower left panel.

In a previous study conducted by one of the authors (Shinba, 2017), it was reported that autonomic responses induced by a mental task differ between patients with MDD and healthy people. Using this difference in transient autonomic responses, we developed a system that enables objective MDD screening without the need for medical history taking (Sun et al., 2016; Dagdanpurev et al., 2018). On the basis of the differences in autonomic responses between schizophrenia patients and healthy controls, we conducted the present study using yoga as the task, with a wearable ECG monitor.

Time series RRIs measured by unipolar precordial ECG of the wearable device were detected using an R-wave peak detection algorithm. The time series of RRIs were interpolated using a spline interpolation and resampled at 4 Hz. The resampling procedure was critical for achieving equal interval sampling for FFT. The power spectrum of the resampled RRIs was calculated using short-time FFT with a 30-s data window and sliding the window by every 2 s. The power spectrum was integrated from 0.04 to 0.15 Hz to determine the LF component of HRV, which is an index of sympathetic and parasympathetic activity, and from 0.15 to 0.4 Hz to determine the HF component of HRV, which is an index of parasympathetic activity (Malik et al., 1996). The very low frequency (VLF; 0.0033–0.04 Hz) component of HRV, which is an index of predominantly sympathetic activity, was determined using the maximum entropy method for a 5-min data window (sliding every 4 s). Time domain HRV indices, SDDN and RMSSD were also calculated which reflects total power of HRV and vagal tone activity, respectively. Four explanatory variables were determined based on *p*-values of Friedman test (VLF, LF, HF, HR, SDNN, and RMSSD) conducted for pre-rest, during yoga, and post-rest in patients with schizophrenia and heathy volunteers.

Artifacts and extra beats assume influence on FFT power spectrum drastically. We conducted following data processing to avoid these artifacts and extra beats. In the case of missing an R-wave induced by artifacts or auriculoventricular block, we put an R-wave at the midpoint of an R-wave missing adjacent R-waves. In the case of extra beats, we eliminated R-waves with RR-interval less than a threshold (such as 400 ms).

### Algorithm development

The proposed system adopted LDA to distinguish the patient group with schizophrenia from the healthy volunteer group. Within these two groups, we defined the following linear discriminant function to minimize within-group variance and maximize intergroup variance:

$$Z=\overset{12}{\underset{n=1}{\sum }}\begin{array}{c} A_{n}X_{n}\;＋\;constant \end{array}$$ (1)

The proposed system classifies an examinee as having suspected schizophrenia when the Z-score is greater than or equal to zero. A_*n*_ (*n* = 1–12) are regression coefficients that correspond to X_*n*_ (*n* = 1–12), which are autonomic nervous activity-related explanatory variables as shown in Figure 1. When the Z-score is greater than or equal to zero, the proposed system classifies the examinee as having suspected schizophrenia, and when the Z-score is less than zero, the proposed system classifies the examinee as being mentally healthy. We used VLF, LF, HR, and SDNN as four explanatory variables.

### Statistical analysis

The ideal combinations of regression coefficients and the constant of LDA were determined by StatMate III (3B Scientific, Tokyo) statistical software using the twelve variables (i.e., means of VLF, LF, HR, and SDNN, pre-rest, during yoga and post-rest derived by the wearable wireless ECG with unipolarprecordial lead).

Using LDA, we evaluated whether the calculated Z function of LDA could discriminate patients with schizophrenia from normal control subjects. The Mahalanobis squared distance and the classification error rate were calculated from the non-contact derived variables, where the Mahalanobis squared distance is an index of the extent to which the discriminant function discriminates between criterion groups (McLachlan, 2004).

The results from the LDA classification model were used to calculate sensitivity, specificity, negative predictive value (NPV), and positive predictive value (PPV). The Chi-squared test revealed that there were no significant differences in the male/female ratio between the schizophrenia and healthy volunteer groups (*p* = 0.15) and no significant differences in age composition when the samples were divided into two generations (subjects younger than 40 years, subjects equal to or older than 40 years) in the schizophrenia and healthy volunteer groups (*p* = 1.00).

### Yoga protocol as a workload

We adopted a 15-min mindfulness-based yoga program for schizophrenia (MYS; for the video, see https://youtu.be/c4xzmA22h84). MYS is a modification of mindfulness-based stress reduction (MBSR) and mindfulness-based cognitive therapy (MBCT). MBSR and MBCT incorporate the essence of traditional yoga meditations and movements that focus on breath and bodily senses (Kimura, 2017). The simple MYS protocol used in the current study was designed to be safe for patients with schizophrenia. Mindfulness and yoga have been widely offered within psychiatric occupational therapy and day care services to alleviate schizophrenia symptoms (Chugh-Gupta et al., 2013; Grensman et al., 2018). Participants were instructed to sit on a chair during the pre-yoga rest period, the 15-min yoga intervention, and the post-yoga rest period. The MYS protocol comprises the three following components: 1) 2 min of mindfulness meditation: breathing corresponds to an awareness of natural breathing; 2) 8 min of meditative movement: two techniques of isometric yoga that repeats “muscle tension” and “relaxation” interlocked breathing with sounds, such as “Ah....“; and 3) 5 min of breathing exercise: two breathing techniques (humming breathing and 1:2 breathing) (Oka et al., 2018).

### General health questionnaire-28

We used the GHQ28 score as a reference, which is a World Health Organization standard general mental health survey score proposed by Goldberg (Goldberg and Blackwell, 1970). The GHQ28 score comprises the four factor scales (i.e., somatic symptoms, anxiety and insomnia, social dysfunction, and severe depression). There are seven questions in each category, resulting in a total of 28 questions. Higher scores indicate lower general mental health status. Those with a GHQ28 score greater than 11 are suspected to have schizophrenia (Fukunishi, 1990).

## Results

**FIGURE 3:**
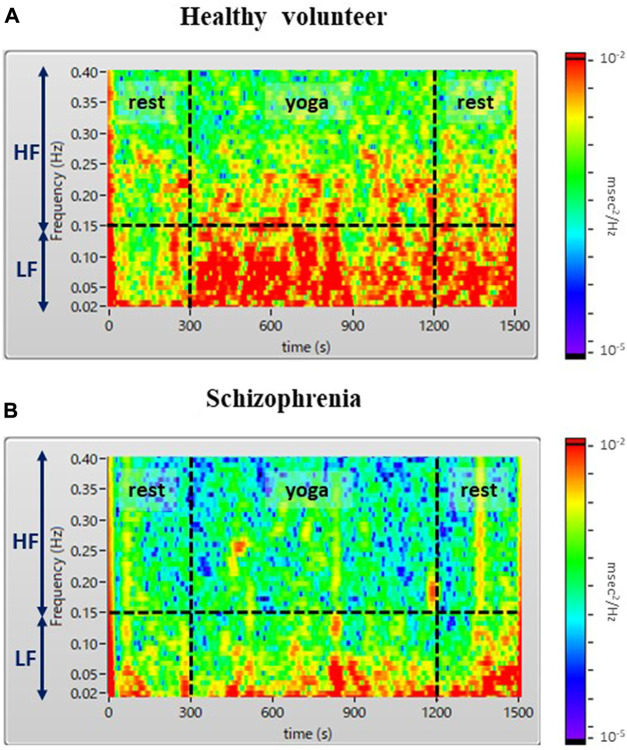
Color maps of a healthy volunteer **(A)** and a patient with schizophrenia **(B)**. The color map shows a drastic yoga-induced increase of power spectrum densities corresponding to the LF and HF domains for the healthy volunteer **(A)**, whereas that of the patient with schizophrenia **(B)** did not show any distinctive yoga-induced changes.

**FIGURE 4:**
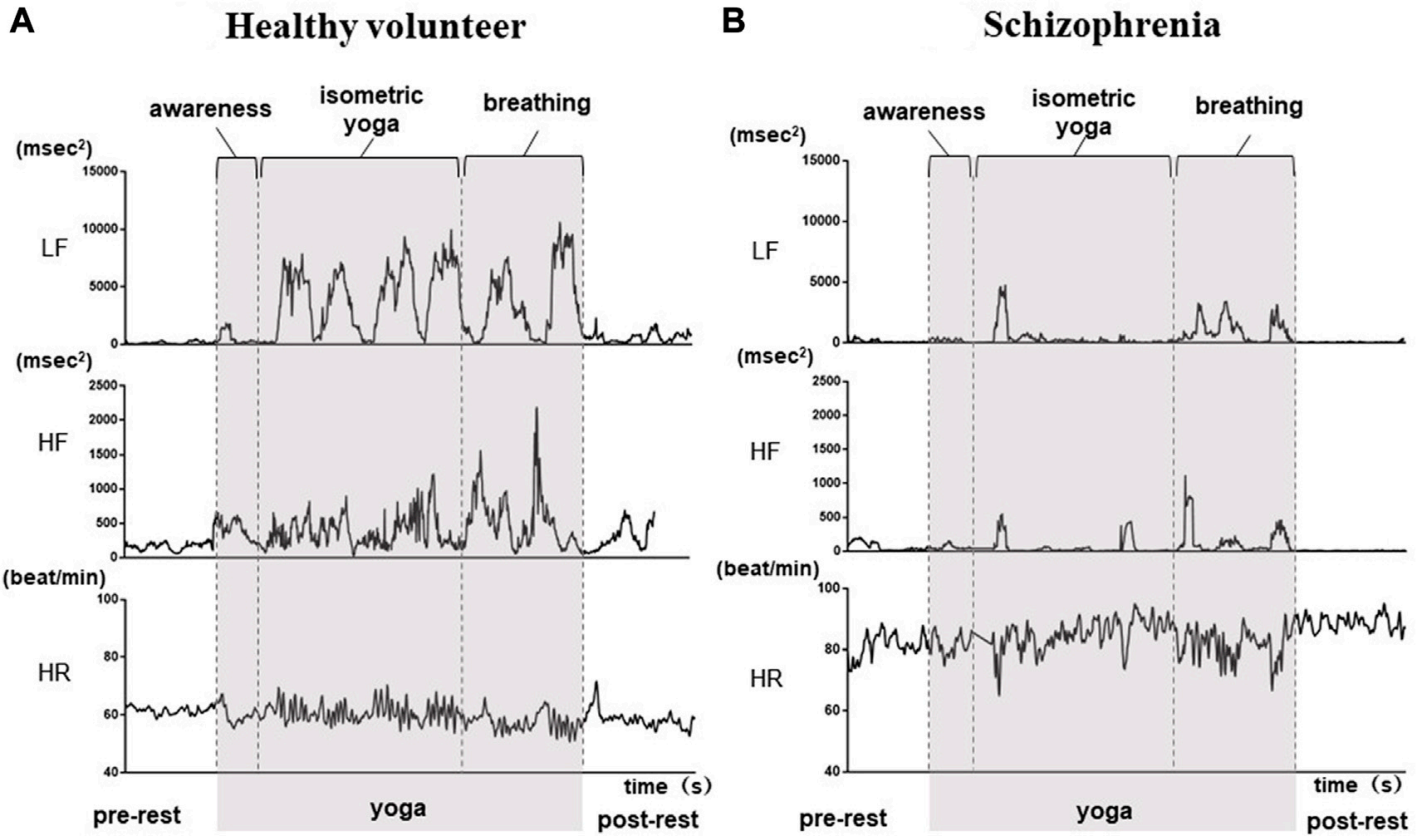
Autonomic response-related parameters (LF, HF and HR) of a healthy volunteer **(A)** and a patient with schizophrenia **(B)** during the pre-rest, during yoga, and post-rest periods. In the healthy volunteer, LF and HF increased drastically during yoga, while those of the patient with schizophrenia did not show distinctive changes during yoga. HR at rest was higher in the patient with schizophrenia compared with that in the healthy volunteer.

The demographic characteristics of the schizophrenia patients and healthy volunteers are shown in Table 1. No significant differences were observed in VLF, LF, HF, HR, LF_n_, HF_n_ SDNN, and RMSSSD at pre-rest. As shown in Table 2, *p*-value of Friedman test conducted for pre-rest, during yoga, and post-rest in patients with schizophrenia, we determined VLF (*p* = 0.10), LF (*p* = 0.0001), HR (*p* < 0.05), and SDNN (*p* = 0.001) as explanatory variables of LDA. HF with the highest *p*-value (*p* = 0.3) in frequency-domain-HRV and RMSSD showing higher *p*-value (*p* < 0.05) than SDNN in time-domain-HRV were excluded. Figure 2 shows a display screen of the proposed system, with yoga-induced power spectrum density mapping of RR intervals corresponding to the pre-rest, yoga practice, and post-rest periods of a healthy volunteer (left) and a patient with schizophrenia (right). The color maps of a healthy volunteer revealed drastic yoga-induced increases in power spectrum densities corresponding to the LF and the HF frequency domains (Figure 3A), whereas those of the patient with schizophrenia did not show drastic yoga-induced changes (Figure 3B). The HR of a patient with schizophrenia at rest was higher than that of a healthy volunteer (Figure 4, upper). The LF and the HF of a healthy volunteer increased drastically during yoga (especially during isometric yoga and breathing exercises), whereas the patient with schizophrenia did not show any distinctive changes during yoga (Figure 4, middle and lower).

**TABLE 1:** Characteristics of schizophrenia patients and healthy volunteers.

	Schizophrenia	Healthy volunteer	
	*n* = 12	*n* = 16	χ^2^ or *t*
Ages (years)	41.3 (10.3)	40.7 (8.7)	*t* = 0.15 n.s.
Sex			
Male	4 (33.3%)	9 (56.2%)	χ^2^ = 1.44 n.s.
Female	8 (66.7%)	7 (43.8%)	
Age at onset	20.1 (6.1)	—	—
Duration of Untreated Psychosis	3.1 (4.2)	—	—
Duration of the disease	19.2 (11.7)	—	—
Chlorpromazine equivalent doses (mg)	561.4 (523.6)	—	—
Pre–rest HR (beat/min)	79.7 (9.9)	72.3 (9.1)	*t* = 1.9 n.s.
Pre–rest VLF (msec^2^)	962.4 (914.2)	1,306.7 (451.0)	*t* = −1.1 n.s.
Pre–rest LF (msec^2^)	356.1 (389.2)	742.2 (826.9)	*t* = −1.5 n.s.
Pre–rest HF (msec^2^)	207.2 (266.2)	323.7 (437.9)	*t* = −0.8 n.s.
Pre–rest LF_n_ (nu)	0.61 (0.20)	0.60 (0.18)	*t* = 0.04 n.s.
Pre–rest HF_n_ (nu)	0.38 (0.20)	0.39 (0.18)	*t* = −0.04 n.s.
Pre–rest SDNN (ms)	38.9 (20.1)	46.1 (15.4)	*t* = 0.9 n.s.
Pre–rest RMSSD (ms)	27.5 (31.1)	29.8 (14.9)	*t* = 0.2 n.s.
GHQ28 score			
Somatic symptoms	3.2 (2.5)	2.3 (1.3)	*t* = 1.1 n.s.
Anxiety/insomnia	3.9 (2.4)	1.6 (1.8)	*t* = 2.5*
Social dysfunction	2.1 (2.0)	0.7 (1.3)	*t* = 2.0 n.s.
Severe depression	3.0 (3.1)	0.4 (1.2)	*t* = 2.5*
Total	12.3 (8.4)	4.9 (4.7)	*t* = 2.6*

*p < 0.05

Data are expressed as mean values (±SD) or as numbers (percentage) for sex. Age at onset is the age at which psychiatric symptoms were first experienced. Duration of untreated psychosis is the period between onset and first medical examination. Duration of the disease is the period between onset and the present time. The chlorpromazine equivalent is a measure of the strength of antipsychotics on the basis of the amount of chlorpromazine contained. VLF, LF, and HF are very low-frequency (0.0033–0.04 Hz), low-frequency (0.04–0.15 Hz), and high-frequency (0.15–0.4 Hz) components of HRV, respectively. LF_n_ and HF_n_ are normalized LF and HF by total power (LF + HF). SDNN is the standard deviation of the RRI averaged every 5 min. RMSSD is the square root of the mean of the squares of the differences between consecutive adjacent RRI. The General Health Questionnaire-28 (GHQ28) is a mental health survey comprising 28 questions classified into four categories. Chi-squared test revealed that there were no significant differences in the male/female ratio between patients with schizophrenia and healthy volunteers. Students’ *t*-test was adopted to test for significant differences between groups.

**TABLE 2:** Friedman test results for comparison of HRV parameters in pre-rest, yoga, and post-rest.

Schizophrenia						
Friedman test	VLF	LF	HF	HR	SDNN	RMSSD
*p*-value	0.1	0.0001	0.3	0.04	0.001	0.04
*p*-value summary	ns	***	ns	*	**	*
Friedman statistic	4.5	18	2.167	6	9.5	6.167
Healthy volunteer						
Friedman test	VLF	LF	HF	HR	SDNN	RMSSD
*p*-value	0.4	<0.0001	0.9	0.001	0.1	0.06
*p*-value summary	ns	****	ns	***	ns	ns
Friedman statistic	1.625	21.13	0.125	14	3.875	5.375

*p < 0.05, **p < 0.01 ***, *p* < 0.001 ****, *p* < 0.0001. The Friedman test results of schizophrenia patients and healthy volunteers conducted for pre-rest, yoga, and post-rest phases.

**FIGURE 5:**
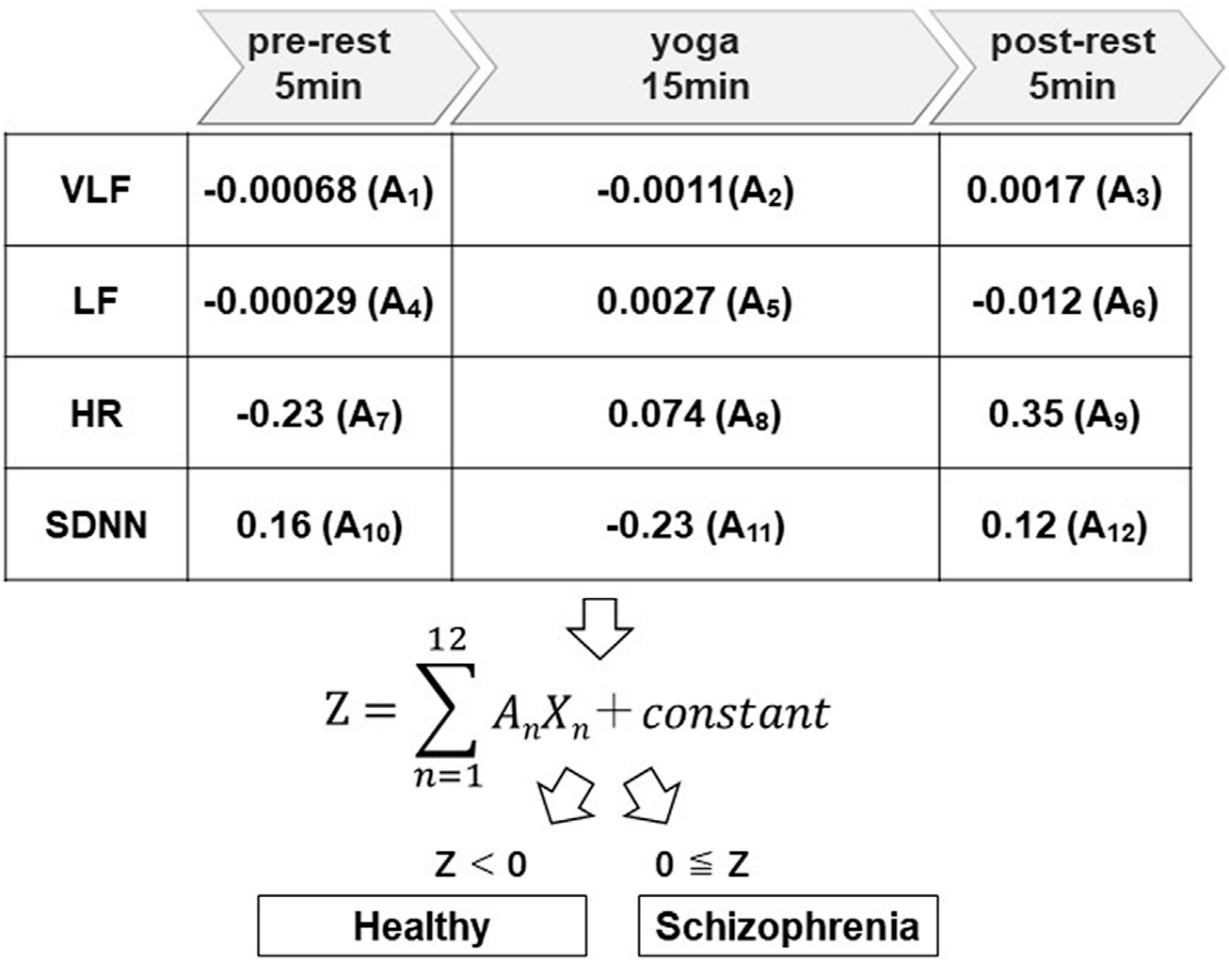
Discrimination algorithms. The regression coefficients (A_1_ to A_12_) were determined using LDA to distinguish patients with schizophrenia from healthy volunteers, where A_1_ to A_12_ are the regression coefficients corresponding to VLF, LF, HR, and SDNN for the pre-rest, during yoga, and post-rest periods.

Using the yoga-induced autonomic responses of 28 participants, we determined the linear discriminant Eq. 2. Twelve explanatory variables as the parameters were adopted for linear discriminant analysis for patients with schizophrenia and healthy volunteers (Total; 24 parameters). The test of normality was conducted with the Shapiro-Wilk test (S-W test), which is applicable even when the sample size is small. 5 out of 24 parameters (Schizophrenia; VLF (pre-rest), LF (pre-post), LF (yoga), Healthy; VLF (post-rest), LF (pre-rest) did not follow normal distribution. Nevertheless, the linear discriminant function calculated by StatMate III using these parameters (including non-normal distributed parameters) showed statistical significance (*p* < 0.001). Regression coefficients (A_1_–A_12_) corresponding to autonomic activity-related explanatory variables (X_1_–X_12_) are shown in Figure 5.

$$\begin{array}{ll} Z= & 0.00068\;VLF\;prerest-0.00115\;VLF\;yoga+0.00176\;VLF\;postrest+0.00029\;LF\;prerest+0.0027\;LF\;yoga-0.012\;LF\;postrest\\ & -0.23\;HR\;prerest-0.074\;HR\;yoga+0.35\;HR\;postrest+0.16\;SDNN\;prerest-0.23\;SDNN\;yoga+0.12\;SDNN\;postrest-12.89 \end{array}$$ (2)

**FIGURE 6:**
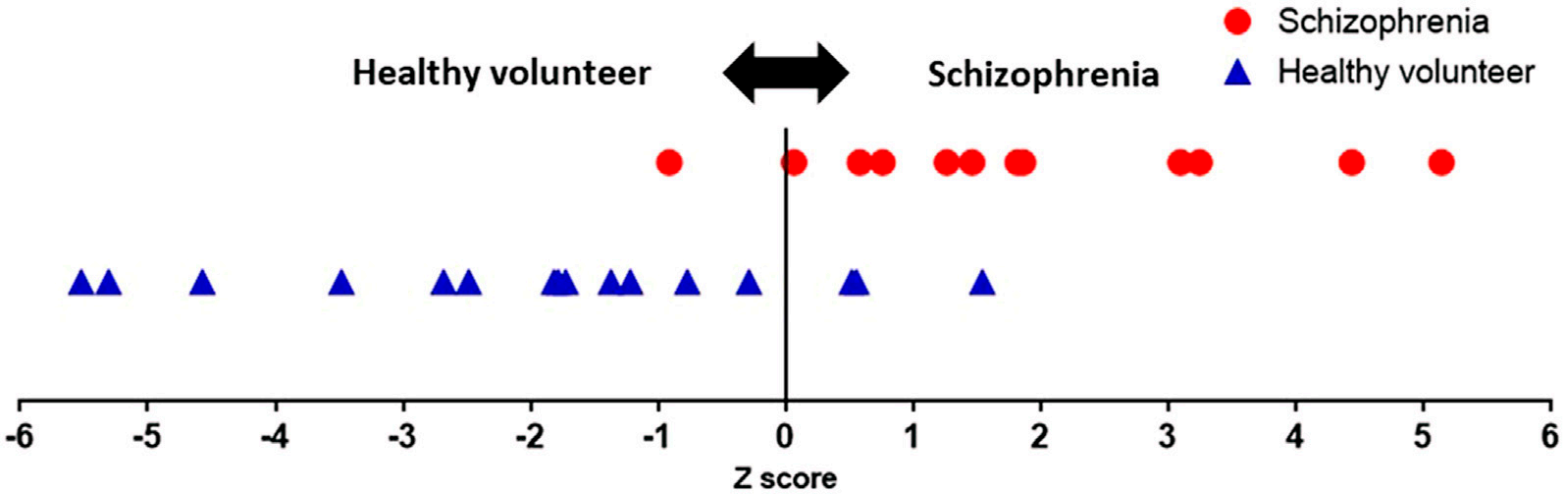
| Z-scores of all participants. A user is suspected to have schizophrenia when the Z-score is greater than or equal to zero. The proposed system achieved sensitivity of 91% and specificity of 81%.

In Eq. 2, Z ≥ 0 indicates suspected schizophrenia, and Z < 0 indicates no suspected schizophrenia. Using this linear discriminant Eq. 2, the Z-scores of the 29 participants were calculated, as shown in Figure 6. The proposed system achieved sensitivity of 91%, specificity of 81%, NPV of 92%, and PPV of 78%. The explanatory variable standardized partial regression coefficients indicate the contribution of each explanatory variable in the linear discriminant function. If we arrange them in order of contribution, SDNN (yoga), LF (post-rest), HR (post-rest), HR (pre-rest), LF (yoga), SDNN (post-rest), SDNN (pre-rest), VLF (post-rest), HR (yoga), VLF (yoga), VLF (pre-rest), and LF (pre-rest). Linear discriminant function adopting top five contribution of explanatory variables revealed 75% sensitivity, 75% specificity, 80% NPV, and 69% PPV. Linear discriminant function showed the highest sensitivity and specificity when all of twelve explanatory variables are adopted. The Mahalanobis squared distance calculated from the HRV-related variables was 3.8. The classification error rate corresponding to the Mahalanobis squared distance was 16.4%. There was no significant correlation between chlorpromazine equivalent doses of antipsychotics and Z-scores (*r* = −0.28, *p* = 0.37).

**FIGURE 7:**
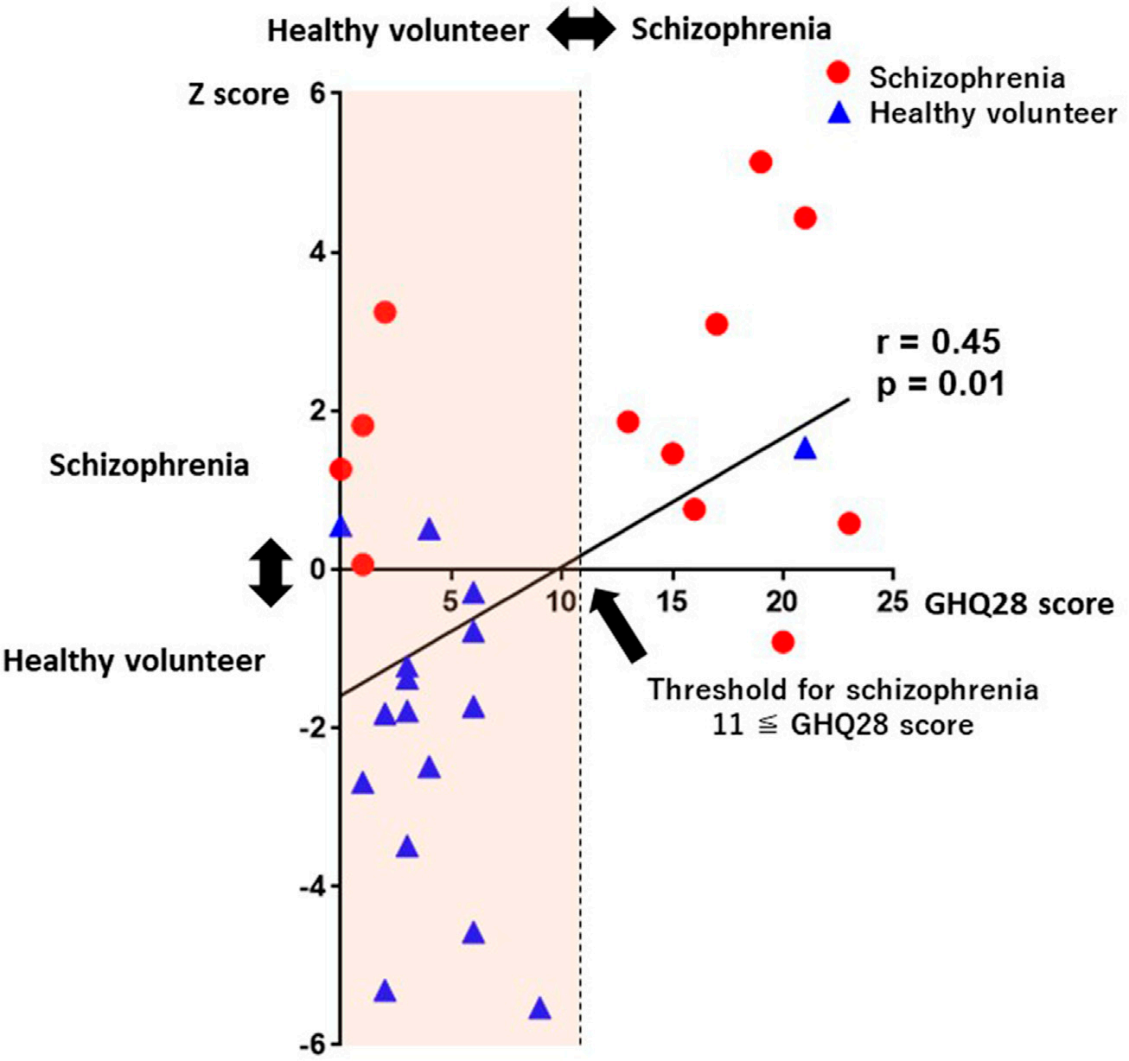
A scatterplot between Z-scores and GHQ28 scores. GHQ28 scores were significantly correlated with Z-scores (*r* = 0.45, *p* = 0.01). Abbreviations: GHQ28, General Health Questionnaire-28.

GHQ28 Total scores were weakly correlated with Z-scores which were determined without history taking (*r* = 0.45, *p* = 0.01; Figure 7). The proposed system not only achieved schizophrenia screening, but also Z-score-based schizophrenia severity detection.

**FIGURE 8:**
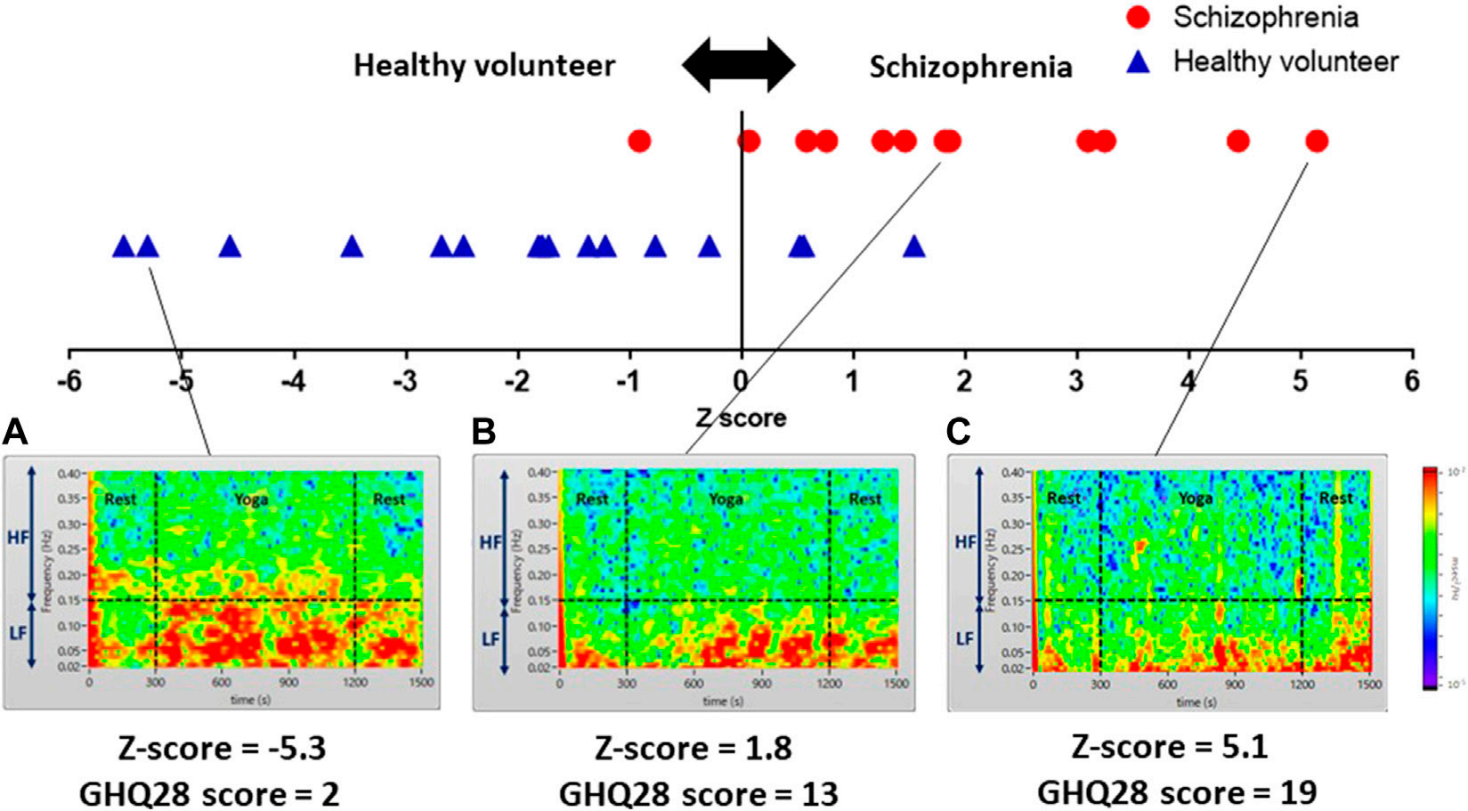
Color maps showing yoga-induced autonomic nervous responses of a healthy volunteer presenting minor symptoms **(A)**, and patients with schizophrenia presenting moderate symptoms **(B)**, and severe symptoms **(C)**. Color maps of a healthy volunteer and patients with schizophrenia visualizing power spectrum densities corresponding to the LF (0.04–0.15 Hz) and HF (0.15–0.4 Hz) domains of pre-rest (0–300 s), during yoga (300–1,200 s) and post-rest (1,200–1,500 s): **(A)** a healthy volunteer presenting minor symptoms (GHQ28 score = 2) showing high power spectrum densities in LF and a portion of HF domain continuously; **(B)** a patient with schizophrenia presenting moderate symptoms (GHQ28 score = 13) showing a yoga-induced moderate increase of power spectrum density in LF domain; **(C)** a patient with schizophrenia presenting severe symptoms (GHQ28 score = 19) showing a yoga-induced slight increase of power spectrum density in the LF domain.

In addition to quantitative assessment, we successfully visualized subjects severity on color maps of LF and HF power spectrum densities during yoga. In Figures 8A–C are the color maps of LF and HF power spectrum densities corresponding to healthy volunteers (A) and patients with schizophrenia(B) (C) presenting minor symptoms (GHQ28 score = 2), those presenting moderate symptoms (GHQ28 score = 13), and those presenting severe symptoms (GHQ28 score = 19), respectively.

## Discussion

Using yoga-induced autonomic nervous activity responses, the current study revealed that patients with schizophrenia could be distinguished from healthy volunteers with sensitivity of 91%, specificity of 81%, NPV of 92%, and PPV of 78%. The Z-score obtained by the proposed system was significantly correlated with the GHQ28 score, which reflects the severity of mental disorder. The correlation coefficients between chlorpromazine equivalent doses of antipsychotics and Z-scores (*r* = −0.28, *p* = 0.37) suggested that chlorpromazine equivalent doses of antipsychotics had no impact on Z-scores. Autonomic activity (LF and HF) in patients with schizophrenia did not show any distinct changes during yoga, whereas the LF and HF autonomic activity of healthy volunteers showed drastic increases. A meta-analytic review of 34 studies reported that parasympathetic activity was reduced in patients with schizophrenia (Clamor et al., 2016). In addition to autonomic activity at rest, we measured transient autonomic nervous activity induced by yoga, because autonomic activity at rest differs substantially between individuals, meaning that HRV parameters (pre-rest) by itself cannot be used for schizophrenia screening. According to the results of Friedman test in Table 2, SDNN, only of patients with schizophrenia, reflecting total power (VLF + LF + HF) of HRV showed statistically significant *p*-value (*p* = 0.001) of Friedman test conducted for pre-rest, during yoga, and post-rest (healthy volunteers, *p* = 0.1). This can be attributed to lower Friedman’s *p*-value of VLF (the lowest part of Total Power) in schizophrenia patients (*p* = 0.1) than that of healthy volunteers (*p* = 0.4). In addition, RMSSD, only of patients with schizophrenia, reflecting vagal tone activity indicated significant *p*-value (*p* < 0.05) of Friedman test (healthy volunteers, *p* = 0.06). Similar symptom was observed in HF, which also reflected vagal tone activity (patients with schizophrenia, *p* = 0.3, healthy volunteers, *p* = 0.9).

On the other hand, frequency domain HRV, especially, HF revealed that yoga did not cause normal stress-induced autonomic responses in patients with schizophrenia. In contrast, Castro et al. reported that autonomic responses to the mental arithmetic stress test were normal in patients with schizophrenia (Castro et al., 2008). The proposed system achieved a high level of screening sensitivity (91%) and specificity (81%), without the need for taking a full medical history.

Previous studies have investigated the potential of various biomarkers for schizophrenia, such as NIRS and EEM testing. NIRS monitors changes in hemoglobin concentration in the prefrontal cortex using near-infrared light reflection and has an accuracy of 69% in schizophrenia screening (Koike et al., 2014). EEM can successfully discriminate patients with schizophrenia from those without schizophrenia with sensitivity of 73% and specificity of 70% (Suzuki et al., 2009). However, these screening systems are expensive, and are not designed for home use. In contrast, our proposed system was designed for home use, although the present study was conducted at hospitals and clinics. The proposed system only requires a wearable device involving a smartwatch for heartbeat monitoring, and a yoga instruction video. Clinical use of proposed system generates cost, but it is expected to be small in cost.

When all the ages were included, Chi-squared test for sex differences did not yield a significant difference (χ^2^ = 1.44 (*) *p* = 0.22), but in the 20–39 age group, 10 males (2 patients with schizophrenia and 8 healthy volunteers) and 4 females (4 patients with schizophrenia and 0 healthy volunteers), there was significant difference (χ^2^ = 7.46 *p* = 0.0062). A statistical imbalance occurred for a sex between the schizophrenia and the healthy volunteer groups.

In addition, because the display screen of the proposed system was designed for medical professionals, it contains an inappropriate amount of clinical information for home use. Thus, for home use, the presented information should be refined to simply recommend that the user visits a psychiatrist if Z ≥ 0. This screening system was not designed to provide a formal diagnosis of schizophrenia. Rather, the proposed system enables quantitative evaluation of schizophrenia severity and visualization of yoga-induced autonomic nervous activation. Though the proposed system cannot exclude false positive schizophrenia, it is designed to encourage a potential patient with schizophrenia to go to a psychiatry.

The level of specificity (91%) and NPV (92%) of the proposed system is relatively. One limitation of this study is that sample size is small. Unfortunately, under COVID-19 pandemic, clinical study at psychiatric outpatient unit was strictly restricted.

On the other hand, we must admit that the present system may not yet be suited for practical use. The wearable ECG adopted for HRV monitoring in this study can be substituted with a smart watch. In the future, the combination of a smartwatch (ECG monitor) and a smartphone (HRV calculation and watching yoga instruction-video online) may enable automated screening without the help of healthcare professionals at home.

Early detection of schizophrenia is important because patients with schizophrenia receive extremely low treatment satisfaction (Japan Health Sciences Foundation, 2019). In addition, a standard biomarker for schizophrenia has not yet been established. In recent years, social withdrawal has become a significant problem (Kato et al., 2019), particularly for cases in which communication with the patient is not maintained because they are staying at home for long periods. In a study of the Japanese population, hikikomori has been reported to account for 30% of patients with schizophrenia experiencing hallucinations, delusions, emotional flattening, and decreased motivation (Tateno et al., 2012). To encourage individuals with suspected schizophrenia to attend a clinical psychiatrist, we developed a novel system that enables schizophrenia screening at home, in which patients practice yoga in accordance with an instruction video while wearing a wearable device involving a smartwatch. Previous reports have revealed that HRV parameters can be measured using a smartwatch with the same precision as those measured by ECG (Hernando et al., 2018; Elgendi and Menon, 2019).

In the context of the worldwide coronavirus disease 2019 pandemic, our proposed system could be used for home-based schizophrenia screening without the risk of infection. This system holds promise for future home-based schizophrenia screening without medical history taking by psychiatrists, and requires only a smartwatch and a yoga instruction video.

## Data Availability

The raw data supporting the conclusion of this article will be made available by the authors, without undue reservation.
